# Pilot Study Comparing the In Vitro Response of Circulating Monocytes to *Aspergillus fumigatus* Swollen Conidia in Patients with Chronic Graft-Versus-Host Disease and Healthy Volunteers

**DOI:** 10.3390/jof11060444

**Published:** 2025-06-11

**Authors:** Claire Kenny, Charles Oliver Morton, Eibhlin Conneally, Ann Atzberger, Anthony Davies, Hermann Einsele, Juergen Loeffler, Thomas R. Rogers

**Affiliations:** 1 Sir Patrick Dun Research Laboratory, Department of Clinical Microbiology, Trinity College Dublin, St. James’s Hospital, D08 NHY1 Dublin, Ireland; clairekenny@mater.ie (C.K.); rogerstr@tcd.ie (T.R.R.); 2 National Stem Cell Transplant Unit, Department of Haematology, St. James’s Hospital, D08 NHY1 Dublin, Ireland; conneale@tcd.ie; 3 Trinity Translational Medicine Institute, Trinity College Dublin, St. James’s Hospital, D08 NHY1 Dublin, Ireland; ann.atzberger@gmail.com (A.A.); anthonmitchelldavies@gmail.com (A.D.); 4 Department of Internal Medicine II, University Hospital Wuerzburg, 97080 Wuerzburg, Germany; einsele_h@ukw.de (H.E.); loeffler_j@ukw.de (J.L.)

**Keywords:** monocytes, *Aspergillus fumigatus*, aspergillosis, host–pathogen interaction, immune dysregulation

## Abstract

Invasive fungal disease (IFD) is a recognised and potentially life-threatening complication of chronic graft-versus-host disease (cGVHD) and its treatment. Invasive aspergillosis (IA), most often due to the species *Aspergillus fumigatus*, is the leading IFD in this setting. IA can occur during the early weeks following allogeneic haematopoietic stem cell transplantation (HSCT) coinciding with profound neutropenia, but increasingly, cases of IA occur after engraftment, coinciding with the occurrence of cGVHD. Immunomodulatory treatments of cGVHD can impair innate immune responses to inhaled *Aspergillus* conidia, increasing the risk of developing IA. Here, in a pilot study, we present an analysis of the phenotypic characteristics (phagocytic efficiency, fungal killing, and cytokine release) of circulating monocytes derived from patients with cGVHD compared to healthy volunteers. We found that there was no statistically significant difference in their ability to phagocytose *A. fumigatus* conidia, and while there was a trend in their reduced ability to kill conidia, this was not significant when compared to the ability of volunteers’ monocytes to do so. Although we could not demonstrate in this small cohort of patients with cGVHD that monocytes may be a factor in the increased susceptibility to IA, further investigation of larger numbers of study subjects is warranted so that in vitro biomarkers may be developed for immune responses to *Aspergillus* in patients with cGVHD.

## 1. Introduction

Invasive aspergillosis (IA) is primarily caused by the ubiquitous opportunistic fungus *Aspergillus fumigatus* and is a major infectious complication of allogeneic haematopoietic stem cell transplantation (allo-HSCT) [1]. It is estimated that an individual may inhale several hundred spores daily, which pass readily into the sinuses and the lung alveoli, from where the majority of infections originate [2]. In both HSCT and solid organ transplant (SOT) recipients, *A. fumigatus* is the most prevalent *Aspergillus* sp. isolated from cases of IA (37.2 to 56% in HSCT and 61.7% in SOT) [1,3,4]. Mortality rates due to IA continue to be high in spite of the currently available antifungal therapies [5]. It is now recognised that in addition to there being a high risk of IA associated with the post-HSCT neutropenic period, there is an additional peak in incidence after discharge from hospital in patients who are being treated for chronic graft-versus-host disease (cGVHD) [6]. This condition generally occurs between 3 months and 24 months following transplantation [7], and develops in 30–70% of allograft recipients, depending on the conditioning regimen [8,9,10]. Corticosteroids are generally used as a first-line treatment of cGVHD [11], and may be used in combination with calcineurin inhibitors [12] or other immunomodulators. Patients receiving these immunosuppressive regimens are generally given long-term mould-active triazole prophylaxis [13], although this strategy is not wholly effective at preventing IA.

Innate and adaptive immune responses have recognised roles in host defence against IFD, and there is a growing appreciation of the fact that cells of the innate immune response play a key role in the early stages of the pathogen–host interaction [14]. The innate immune system of the immunocompetent individual usually works efficiently to clear inhaled *Aspergillus* spores [15]. Circulating monocytes represent one of the major categories of professional antigen presenting cells (APCs), which play an important role in linking the innate and adaptive immune responses [16]. It is evident from experimental models that monocytes mobilise from the circulation to the lungs in response to pulmonary *Aspergillus* infection. These monocytes can inhibit conidial germination, kill ingested conidia, and release pro-inflammatory cytokines [17]. Monocytes also differentiate into dendritic cells and macrophages, thereby broadening the scope of their role in host defence [18].

Low monocyte numbers and severe GVHD were identified as factors significantly associated with development of IA [19]. As a consequence of that study, examining functions of monocytes, e.g., phagocytosis and cytokine release, after exposure to fungal pathogens may provide an additional form of post-transplantation monitoring that can indicate the risk of developing IA. Monocytes are abundant and relatively easy to isolate from blood, providing a potentially valuable resource for determining host susceptibility to IA.

We conducted a pilot study on the interaction between *Aspergillus fumigatus* and circulating monocytes in patients with cGVHD to see if the phenotypic characteristics of patients’ cells differed from healthy controls. We hypothesised that any identified deficiency in phagocytosis, fungal killing, or cytokine release could represent a risk factor for IA in individual patients with cGVHD. It was not the aim of this study to explain the role of monocytes in susceptibility to IA.

## 2. Materials and Methods

### 2.1. Patients

Patients aged 28–55 years with a diagnosis of moderate or severe cGVHD following allo-HSCT for haematological malignancies [20], who were more than 3 to 9 months post-transplantation and receiving ongoing steroid immunosuppressive therapy +/− other cGVHD therapies, were eligible for inclusion (Table 1). Most of these patients were treated with prednisolone, tacrolimus, or both. The blood monocyte counts at the time of sampling ranged from 0.2 × 10^9^ per mL to 0.9 × 10^9^ per mL. The blood samples had been examined post-HSCT for evidence of chimerism and, although this was confirmed in all cases, the extent of chimerism (% donor vs. recipient cells) was not collected for this study. This study was part of a larger research project investigating biomarkers for the early detection of IA, and was approved by the local St James’s Hospital Research Ethics Committee (Approval Reference 2009/29/04). After obtaining patients’ informed consent, blood was collected for the isolation of circulating monocytes and later cytokine studies, and also from healthy volunteers, who gave their consent prior to their participation.

### 2.2. Isolation of Monocytes from Patient and Volunteer Blood

A modification of the method of Serbina et al. (2009) was used [21]. Peripheral blood mononuclear cells were separated from 30 mL of EDTA-anticoagulated blood from healthy volunteers and cGVHD patients via Ficoll-Hypaque density gradient centrifugation (Axis-Shield, Dundee, UK). Monocytes were isolated from PBMCs using MACS^®^ Technology (Miltenyi Biotech, Bergisch Gladbach, Germany) via magnetic labelling of the CD14 receptor and subsequent magnetic cell sorting (Miltenyi Biotech). In brief, monocytes were labelled with CD14 antibodies conjugated to fluorescein isothiocyanate (FITC) (Miltenyi Biotech) as per the manufacturer’s instructions. The binding of CD14 antibody to the CD14 receptor does not initiate signal transduction, as the CD14 receptor lacks a cytoplasmic domain (Technical notes, Miltenyi Biotech). For the co-culture experiments, the monocytes were resuspended in RPMI-10 [RPMI (GIBCO/BRL, Grand Island, NY, USA) + 10% heat-inactivated FCS (GIBCO-BRL) + penicillin (100 units/mL) + streptomycin (0.1 mg/mL)] at a final concentration of 5 × 10^5^ per mL. The viability of the purified monocytes was analysed using 50 µM Sytox Blue nucleic acid stain (Thermo Fisher Scientific, Waltham, MA, USA). In Figure S1, the purity and viability of the CD14+ cells were confirmed to be around 90%. Not all patient samples were used in further experiments due to insufficient yield.

### 2.3. Aspergillus fumigatus Cultivation and Fluorescent Labelling

*Aspergillus fumigatus* clinical isolate AF293, which has been widely researched in host–pathogen interaction studies, was used for all experiments. The fungus was grown, and co-culture experiments were performed in a BSL2 containment facility that was compliant with the local biosafety requirements. It was grown on malt agar at 37 °C for 3 days and then harvested. To remove clumps and spore heads, the conidia were filtered twice through 40 µm nylon cell strainers (BD Falcon, Franklin Lakes, NJ, USA). To generate swollen conidia, they were resuspended in RPMI (GIBCO-BRL) and incubated overnight at room temperature. For fluorescent staining, the swollen conidia were incubated overnight at 4 °C in the presence of Alexa Fluor 647^®^ succinimidyl ester dye (Molecular Probes, Eugene, OR, USA) according to the manufacturer’s instructions, washed three times in PBS, and resuspended in RPMI-10 at a final concentration of 1 × 10^7^ per mL.

### 2.4. High Content Screening to Quantify How Many Conidia Were Phagocytosed

Following co-culture (multiplicity of infection of 1:2), approximately 1 × 10^4^ FITC-labelled monocytes from each gate were sorted into the wells of a poly-L-lysine-treated 96-well plate. The monocytes were fixed in 4% paraformaldehyde (Sigma-Aldrich, St. Louis, MO, USA), washed 3 times with PBS, and permeabilised for 30 min with 0.1% Triton-X (Sigma Aldrich). The cells were then washed extensively and stained with 0.1 µg/mL Phalloidin-TRITC (Molecular Probes) and 5 µg/mL Hoechst 33342 nuclear DNA stain (Molecular Probes) for 1 h. The wells were washed a further three times before viewing.

### 2.5. Cell Sorting

Cells were sorted on a MoFlo XDP (Beckman Coulter, Brea, CA, USA) using the above-mentioned criteria to select for monocytes with no ingestion and monocytes with ingested conidia.

### 2.6. Confocal Microscopy to Monitor Staining and Phagocytosis

Cells from each gate were sorted onto poly-L-lysine glass slides (Sigma-Aldrich). The cells were then allowed to adhere for 15 min, and then fixed with 4% paraformaldehyde. Ten microlitres of VectaShield^®^ HardSet Mounting Medium (Vector Laboratories, Newark, CA, USA) was applied to the slide, and the cells were then visualised with a Laser Scanning Microscope (Zeiss, Oberkochen, Germany) under 63× magnification.

### 2.7. Quantification of Phagocytosis by Monocytes

Experiments using healthy volunteer or patients’ monocytes were run concurrently. The labelled monocytes and labelled conidia were co-incubated at a multiplicity of infection of 1:2 at 37 °C for 3 h. The interaction lasted for 3 h to maintain consistency with the assay to assess fungal killing. Live swollen conidia transitioned to germ tubes after 3 h; longer incubations would create inconsistencies between the phagocytosis, fungal killing, and cytokine analyses.

For the inhibition of phagocytosis, Cytochalasin D (Sigma-Aldrich) was added at a final concentration of 20 g/mL. The samples were then analysed using a CyAn Flow Cytometer (Beckman Coulter, Brea, CA, USA). Using dual laser excitation at 488 nm and 635 nm, fluorescence emission was collected with a bandwidth filter at 530/40 nm for CD14-FITC and at 660/20 nm for Alexa Fluor 647^®^. Viable monocytes (stained with Hoechst 33342) were gated, and debris and clumps were excluded using the forward scatter (FSC) and side scatter (SSC) parameters. The gate was transferred to a dot plot of FITC versus Alexa Fluor 647^®^. The increase in fluorescence in the Alexa Fluor 647^®^ parameter represented the ingestion of conidia by the monocytes.

### 2.8. Measurement of Fungal Killing by Monocytes

Following co-culture (multiplicity of infection of 1:2), fungal killing by monocytes was measured using a method similar to ones used previously for fungal killing by monocyte-derived dendritic cells [22] and bacterial killing by neutrophils [23]. At 0 h (less than 5 min of co-incubation) and 3 h time points, 100 µL aliquots of co-culture mix were rescued in fresh microfuge tubes containing 900 µL of 0.1% Triton-X (Sigma-Aldrich). Conidia incubated in RPMI-10 medium alone were used as a control. The samples were then vortex-mixed for 10 s to lyse the monocytes. Serial dilutions were performed, plated in duplicate onto malt extract agar plates, and grown for 20 h at 37 °C. The percentage viability of conidia in the monocyte/conidia co-culture mix was then calculated as a percentage of conidia viability in the medium alone.

### 2.9. Measurement of Cytokine Release

Supernatants were collected from the *Aspergillus*–monocyte interactions (co-cultured at a multiplicity of infection of 1:2) at both 0 h and 3 h, and analysed for IL-1 β, IL-6, IL-8, IL-10, and IL-12, Interferon-gamma, and TNF-alpha using the Meso Scale Discovery 7-plex ELISA assay.

### 2.10. Statistical Analyses

Comparisons of phagocytosis and fungal killing between patients and volunteers, and cytokine release, using the amount of cytokine released at 0 h compared to that released at 3 h, were analysed using the Mann–Whitney test in Graphpad Prism (Version 10.4). Correlation analyses between cytokine release and fungal killing were conducted via bivariate analysis using Pearson’s coefficient in SPSS (SPSS Statistics Version 30).

## 3. Results

### 3.1. Measurement of Phagocytosis and Fungal Killing

**Figure 1:**
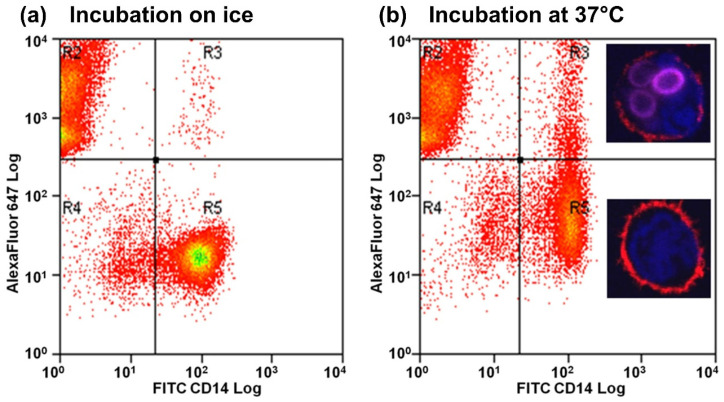
Flow cytometry scatterplots showing the phagocytosis of conidia by donor monocytes. (**a**) Result of phagocytosis assay after incubation for 3 h on ice. On average, the R3 quadrant contained 0.4% and the R5 contained 32.75% of total cells (monocytes and conidia). (**b**) Results of phagocytosis assay after 3 h at 37 °C. On average, the R3 quadrant contained 4.6% and the R5 quadrant contained 25% of total cells (monocytes and conidia). Red in the flow chart indicates low intensity, higher intensity denoted by orange, yellow and green (highest intensity). Micrograph inserts taken after cell sorting show the presence of conidia in the monocytes from the R3 quadrant, whereas monocytes from the R5 quadrant do not contain conidia. Further micrographs confirm phagocytosis of swollen conidia in Figure S2.

Confocal analysis of sorted cells from FACS analysis indicated that monocytes from the R3 quadrant contained stained conidia, whereas those in the R5 quadrant did not contain any conidia (Figure 1). Confocal microscopy of co-incubation experiments confirmed that stained conidia were phagocytosed by both patient and volunteer monocytes (Figure S2).

**Figure 2:**
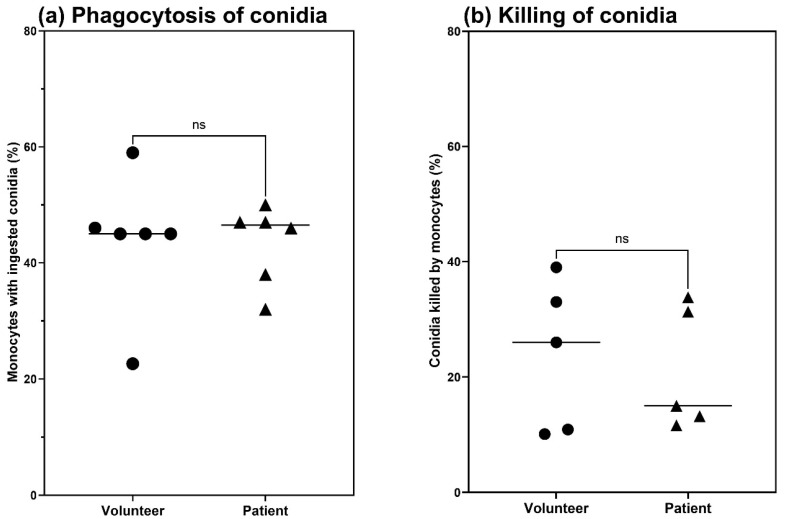
Comparison between phagocytosis (n = 6, for both volunteers and patients) and killing of conidia (n = 5 for both volunteers and patients) based on patients’ and volunteers’ monocytes. (**a**) The percentages of total monocytes ingested by both volunteer and patient monocytes were not significantly different (*p* = 0.6126). (**b**) There was a trend towards volunteers’ monocytes killing more conidia than patients’ monocytes, but this was not a significant difference (*p* = 0.7173). The individual data points and median value (horizontal line) are presented.

FACS analysis showed that the rate of phagocytosis for patient and volunteer monocytes was not significantly different, *p* = 0.6126 (Figure 2a).Comparison of fungal killing by monocytes in patients with cGVHD and healthy volunteers indicated that there was a trend towards greater killing by volunteer cells; however, this was not a statistically significant difference, *p* = 0.7173 (Figure 2b). Indeed, effective fungal killing did not appear to be linked to whether the study subject was a healthy volunteer or a patient with cGVHD. Larger numbers of patients and volunteers would need to be examined to make any definite conclusions on the impairment of fungal killing by monocytes in cGVHD patients.

**Figure 3:**
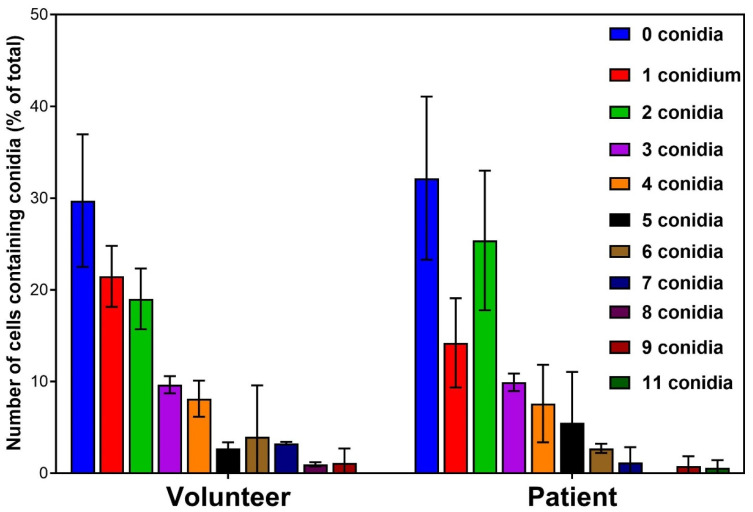
Measurement of the numbers of conidia phagocytosed by monocytes isolated from healthy volunteers and patients with cGVHD. There was no significant difference in the phagocytic capacity of monocytes isolated from volunteers or patients; the ratios of uptake are almost identical for both. The data presented are mean and standard error from three replicates.

High content analysis revealed that both patient and volunteer monocytes ingested similar numbers of conidia, supporting the FACS data (Figure 2a). Each group produced almost identical profiles for the number of conidia ingested (Figure 3).

These data (Figure 2 and Figure 3) indicate that there are no significant differences between patient and volunteer monocytes with regard to phagocytic capacity or fungal killing.

### 3.2. Measurement of Pro-Inflammatory Cytokine Release

Multiplex ELISA analysis (Table 2) measured the induction of cytokine release by conidia. Both patient and volunteer monocytes showed the induction of IL-8, but only volunteer monocytes showed the clear induction of TNF-α after exposure to conidia. Patients’ monocytes had a trend of producing more IFN-γ and IL-10 at the beginning of the experiment. Greater cytokine release at the start of the experiment may be associated with cytokine dysregulation from cGVHD (Table 2) [24]. Based on these data from our small patient cohort, and a limited selection of pro-inflammatory cytokines.

### 3.3. Correlation Between Fungal Killing and Cytokine Release

**Figure 4:**
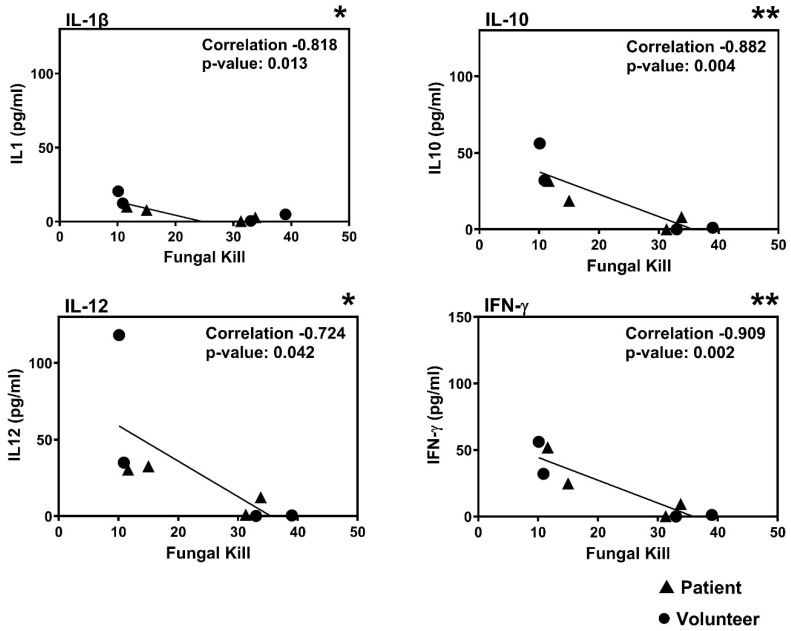
Correlation analysis of the amount of pro-inflammatory cytokines produced by patient cells (triangles) compared to the amount of conidia killed by the volunteer cells (circles). A negative correlation between the release of pro-inflammatory cytokines and fungal killing after interaction between monocytes and *A. fumigatus* conidia for 3 h was observed. The figures indicate that monocytes from individuals that are inefficient at killing *A. fumigatus* conidia produce more IL-1, IL-10, IL-12, and IFN-γ than monocytes that are more efficient at killing the fungus. Equal numbers of patient and volunteer samples were found at each end of the result distribution. Data was analysed via bivariate analysis (Pearson’s coefficient) in SPSS, lines indicate the linear regression of the data points. * *p* < 0.05, ** *p* < 0.01.

There was a trend towards greater fungal killing by volunteer cells, but this was found not to be statistically significant and could easily have been skewed by the small sample size. Correlation analysis was performed to determine if this phenotypic characteristic was related to other properties such as cytokine release. It was found that there was a significant negative correlation between fungal killing and the release of IL-1β, IL-10, IL-12, and IFN-γ after 3 h of co-incubation between monocytes and *A. fumigatus* (Figure 4). The correlation did not reflect the health status of the volunteers, but may be an inherent quality of the cells linked to the individual, and not to cGVHD.

## 4. Discussion

In this study, we measured the phenotypic characteristics of monocytes that had been isolated from blood samples taken from patients with cGVHD and from healthy volunteers, interacting with swollen conidia of *A. fumigatus*. This was conducted in an effort to discover factors that could indicate increased susceptibility to IA. Analysis of phagocytosis via flow cytometry and high content screening did not reveal any significant differences between volunteer and patient monocytes. There was also no significant difference detected in the ability to kill *A. fumigatus* conidia between volunteer and patient monocytes, although the median rate of kill was greater in volunteer monocytes.

The release of pro-inflammatory cytokines was also measured after interaction between monocytes and *A. fumigatus*. Patients’ monocytes showed a significantly greater release of IFN-γ and IL-1β at the start of the experiment (Table 2). IL-1β is one of the primary initiators of inflammation and is normally tightly controlled [25]. Its higher abundance in the supernatant at 0 h of the monocyte–conidia interactions suggests that the patient monocytes came from an environment experiencing potentially damaging excessive inflammation, which is associated with cGVHD. These data for the early induction of cytokines resemble expression analysis that compared monocyte-derived dendritic cells (moDC) and peripheral blood myeloid dendritic cells [26]. There was increased expression of pro-inflammatory cytokine genes in moDC at 0 h. It was hypothesised that this activation was caused by the process required to cause the differentiation of monocytes to moDC. Both patient and volunteer monocytes showed induction of the chemoattractant IL-8, which attracts and activates neutrophils in inflammatory regions, and volunteer monocytes also showed significant induction of TNF-α (Table 2). TNF-α is associated with the immune response to fungal infection, and increased expression has been linked to an augmented capacity of PMNs to damage *Aspergillus* hyphae, possibly through enhanced oxidative mechanisms, and to increased phagocytic activity against conidia [27]. The importance of TNF-α in antifungal immunity has also been revealed by the development of IA in patients being treated with antibodies that block TNF-α [28].

The Th1 response is important for the clearance of fungal infections [29,30,31]. Cytokines indicative of a Th1 response have been measured as up-regulated in response to challenges with *A. fumigatus* [22,32]. The role of the Th1 response in antifungal immunity is further supported by the effect of calcineurin inhibitors on patient outcomes [30,33] and through the effectiveness of IFN-γ as a component of treatment strategies for IA [34]. Data from our study (Table 2) showed significant increases in TNF-α and IL8 after interaction between isolated monocytes and *A. fumigatus* (Table 2). However, IFN-γ and IL-1β showed decreased release in monocytes isolated from patients with cGVHD when exposed to *A. fumigatus* (Table 2). Reduced IFN-γ has been associated with poorer outcomes in murine models of IA. Furthermore, the ratio of IFN-γ to TNF-α was important in the outcome of infection [32]. More TNF-α and less IFN-γ was a characteristic found in mice that failed to clear *Aspergillus* infection, whereas high IFN-γ and lower TNF-α were associated with fungal clearance [32]. The data in this study was only collected over a short period compared to 24 h in the murine study; however, both patient and volunteer samples showed increased release of TNF-α compared to IFN-γ after 3 h (Table 2).

This difference in the TNF-α to IFN-γ ratio could be fungal morphotype-dependent. Our study used resting and swollen conidia, which cannot develop into germ tubes in 3 h, whereas murine studies use conidia that germinate into hyphae in susceptible hosts [32,35]. It has been observed that immune cells are influenced by the presence of immune inert structures such as the rodlet layer RodA or DHN melanin on conidia, and by the exposed antigens on the surface of fungi, and these differ between morphotypes, leading to responses driven through TLR 2 or 4 [36,37]. TLR 4 responses typically result in the Th1 response, whereas a Th2 response was observed in cells activated through TLR 2. It was not possible to measure large differences between cytokines released by patients and volunteers. However, it was observed that the amount of fungal killing was correlated to cytokine release (Figure 4). This is not a general observation about the relationship between cytokine release and fungal killing, as pro-inflammatory cytokines are released in response to *A. fumigatus* [38]. The scale of the response is greater than we observed, because our study only lasted for 3 h. This observation is restricted to comparison between patient and volunteer monocytes under our experimental conditions. Monocytes from individuals who produced less cytokines (IL-1β, IL-10, IL-12, and IFN-γ) had the greatest capacity to kill the fungus, and equal numbers of patient and volunteer samples were in this group (Figure 4). The greater fungal killing suggests a Th1 response, whereas inefficient killing with greater cytokine release suggests a Th2 response (Figure 4 and [29]). Cytokine production may vary as DCs mature, as mature DCs produce less IL-12 compared to immature DCs when stimulated with IL-1β [39]. Changes in cytokine release due to maturation may contribute to the correlation between cytokine concentration and fungal killing (Figure 4). However, the presence of IL-10 during maturation can impair the capacity of DCs to employ a Th-1 response [40] and maturation in other APCs, such as monocytes [41]. The activity of specific cytokines may help to explain the correlation between the IL-10 concentration and fungal killing (Figure 4).

The differences in fungal killing and cytokine release in this study may be related to host genotype. Not all at-risk patients develop IA, which suggests that factors other than direct immunosuppression may contribute to the development of IA, with an incidence of 5–10% in allogeneic HSCT recipients [6,42]. Genotypic factors such as single nucleotide polymorphisms (SNPs) in immunity-related genes have been correlated with an increased susceptibility to IA [43,44]. This suggests that inherent risks to IA are exacerbated by immunosuppression during or post transplantation. However, there has been scarce analysis to confirm the functional relevance of these polymorphisms, and further information is required from detailed genome-wide association studies before SNPs can be reliably employed in prognostic applications [45,46].

This pilot study provides valuable preliminary data that could form the basis for a strategy to assess functional responses of monocytes to *A. fumigatus* in patients with cGVHD. The significant association between low monocyte concentrations and the development of IA supports the focus on monocytes [19]. The value of this research is in potentially identifying if donors and recipients have an increased risk of IA, allowing suitable prophylactic measures to be prepared. This approach has indicated that individual phenotypic measurements of APC may not be useful as the basis for assessing post-transplantation risk of IA. The small number of participants in our pilot study means that further investigations will be necessary. Further studies would need to include a larger study population and assess antigen presentation and other functional aspects of monocytes in immunity to IA. This research could lead to a validated assay for testing the risk of IA during GVHD. Genetic analysis would be needed to determine the basis of the correlation between fungal killing and cytokine release, since this was not related to cGVHD.

## Figures and Tables

**Table 1:** Summary of patients (n = 7) included in the study.

Variable	Detail	Number of Patients
Underlying Condition	Chronic lymphatic leukaemia (CLL)	2
	Mantle cell lymphoma	1
	Myelodysplastic syndrome (MDS)	2
	Acute myeloid leukaemia	2
Treatment	Allogeneic HSCT	6
	HSC transplant + donor lymphocyte infusion at 7 months post-Tx	1
GVHD Severity	Moderate	5
	Severe	2

**Table 2:** Release of inflammatory cytokines (pg/mL) from monocytes, isolated from volunteers and patients with cGVHD, interacting with swollen conidia from *A. fumigatus* over three hours.

	Patient		Volunteer	
Cytokine ^a^	0 h	3 h	0 h	3 h
INF-γ	**37.5 ± 14.6** *^,b^	7 ± 4.8	12.2 ± 6.7	19.6 ± 19.1
TNF-α	18 ± 7.2	28.6 ± 14.3	10.3 ± 3.7	**61.6 ± 20**
IL1β	**9.6 ± 3.8**	2.3 ± 1.5	4.8 ± 1.4	5.7 ± 3.8
IL6	7.8 ± 3	2.9 ± 1.6	6.4 ± 1.8	16.4 ± 5.4
IL8	6.2 ± 2	**178.2 ± 42.1**	5.3 ± 1.6	**299 ± 58.8**
IL10	19.7 ± 8.8	5.4 ± 3.7	6.6 ± 3.9	11.6 ± 11.1
IL12	25.3 ± 8.7	9.3 ± 6.3	28.8 ± 8.9	23.8 ± 23.6

^a^ Cytokines expressed as pg/mL; * bold figures are statistically significant compared to their matched time. ^b^ Mean and standard error presented (n = 5). Statistical analysis performed with Mann–Whitney test.

## Data Availability

The original contributions presented in this study are included in the article/Supplementary Material. Further inquiries can be directed to the corresponding author.

## Supplementary Materials

The following supporting information can be downloaded at: https://www.mdpi.com/article/10.3390/jof11060444/s1. Figure S1, Flow cytometry scatterplots showing (a) viability of donor monocytes and (b) the phagocytosis of conidia by donor monocytes. (a) Uptake of sytox blue is an indicator of cell death; the purified monocytes in quadrant R4 were 87.8% viable. (b) Focusing on the CD14-FITC staining, at least 90% (Quadrants R3 and R5) of the human cells in the experiment were CD14^+^ monocytes. Figure S2, Fluorescence micrographs showing the staining of both monocytes and A. fumigatus conidia. (a) Monocytes isolated from peripheral blood stained with an anti-CD14 FITC antibody. (b) Ethanol inactivated swollen conidia stained with Alexa Fluor 647^®^ succinimidyl ester dye. (c) Merged micrograph showing conidia within the monocytes. (d) Confocal micrograph of monocytes that have interacted with conidia. Some of the monocytes contain phagocytosed conidia. The side bars show that the conidia are present within the monocytes. Monocytes were stained with DAPI (blue) and Phalloidin-TRITC (red). The conidia were stained with Alexa Fluor 647^®^ (magenta).
