# Supplementary figures and images for: Pilot Study Comparing the In Vitro Response of Circulating Monocytes to Aspergillus fumigatus Swollen Conidia in Patients with Chronic Graft-Versus-Host Disease and Healthy Volunteers
Source: J Fungi (Basel). 2025 Jun 11;11(6):444. doi: 10.3390/jof11060444 (PMC12193975; doi:10.3390/jof11060444)

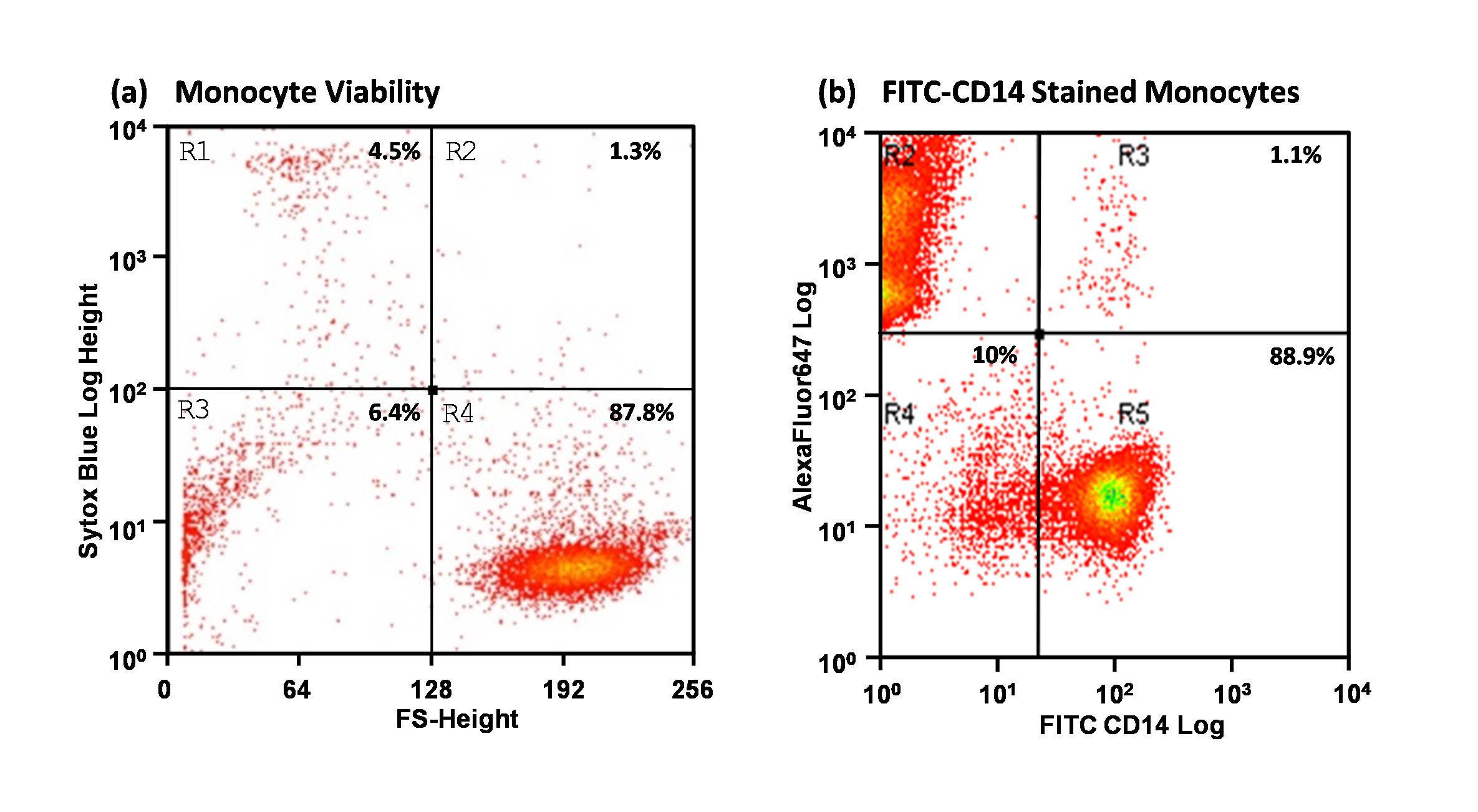

Supplement: Supplementary file 1 [file jof-11-00444-s001.zip › Figure S1.jpg]

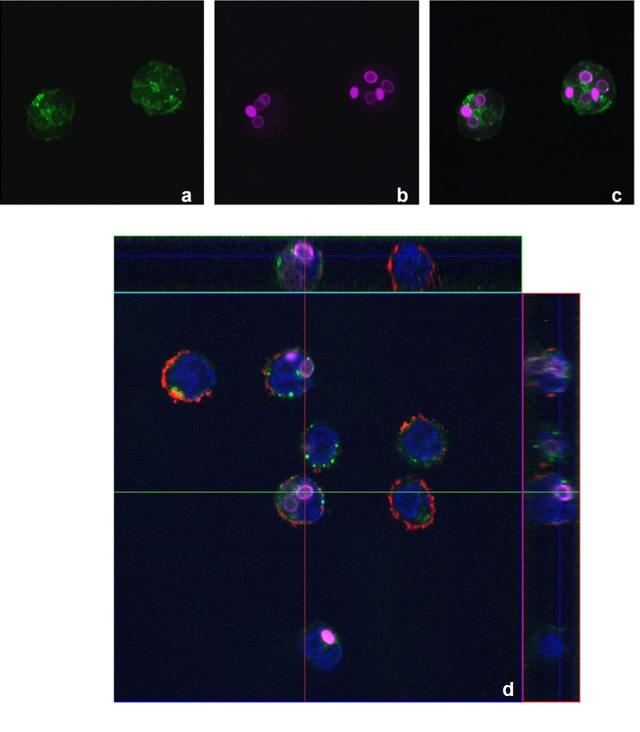

Supplement: Supplementary file 1 [file jof-11-00444-s001.zip › Figure S2.jpg]
